# Hepatitis E Virus (HEV) egress: Role of BST2 (Tetherin) and interferon induced long non- coding RNA (lncRNA) BISPR

**DOI:** 10.1371/journal.pone.0187334

**Published:** 2017-11-01

**Authors:** Daizy Paliwal, Prashant Joshi, Subrat Kumar Panda

**Affiliations:** Department of Pathology, All India Institute of Medical Sciences, New Delhi, India; University of Cincinnati College of Medicine, UNITED STATES

## Abstract

**Background:**

The biology of Hepatitis E Virus (HEV), a common cause of epidemic and sporadic hepatitis, is still being explored. HEV exits liver through bile, a process which is essential for its natural transmission by feco-oral route. Though the process of this polarised HEV egress is not known in detail, HEV pORF3 and hepatocyte actin cytoskeleton have been shown to play a role.

**Methods:**

Our transcriptome analysis in Hepatitis E virus (HEV) replicon transfected Huh7 cells at 24 and 72 hrs indicated that at 24hrs, both LncBISPR and BST2, expressed by a bidirectional promoter were highly upregulated whereas at 72 hrs, BST2 expression was comparatively reduced accompanied by normal levels of BISPR. These findings were confirmed by qPCR analysis. Co-localisation of BST2 and HEV pORF2 was confirmed in HEV transfected Huh7 by confocal microscopy. To investigate the role of BISPR/BST2 in HEV life cycle, particularly virus egress, we generated Huh7 cells with ~8kb deletion in BISPR gene using Crispr-Cas9 system. The deletion was confirmed by PCR screening, Sanger sequencing and Real time PCR. Virus egress in ΔBISPR Huh7 and Huh7 cells was compared by measuring HEV positive strand RNA copy numbers in cell lysates and culture supernatants at 24 and 72 hrs post HEV replicon transfection and further validated by western blot for HEV pORF2 capsid protein.

**Results:**

ΔBISPR Huh7 cells showed ~8 fold increase in virus egress at 24 hrs compared to Huh7 cells. No significant difference in virus egress was observed at 72hrs. Immunohistochemistry in histologically normal liver and HEV associated acute liver failure revealed BST2 overexpression in HEV infected hepatocytes and a dominant canalicular BST2 distribution in normal liver in addition to the cytoplasmic localisation reported in literature.

**Conclusions:**

These findings lead us to believe that BISPR and BST2 may regulate egress of HEV virions into bile *in vivo*. This system may also be used to scale up virus production *in vitro*.

## Introduction

Hepatitis E virus (HEV) is a common cause of both epidemic and sporadic viral hepatitis. The complete biology of this virus is not well understood. The virus egress from hepatocytes occurs through bile canaliculi for feco-oral transmission [1]. The factors involved in this polarized virus egress have been identified as a small viral protein pORF3 [2,3] and hepatocyte cytoskeleton [4]. However, virus replication [5], recycling and cytoplasmic retrotranslocation [6,7] of HEV capsid protein (pORF2) have been described to occur on endoplasmic reticulum.

One of the major bottle-necks in HEV research is production of enough virus in cell culture system for downstream experiments and applications. Although virus egress occurs in HEV infected cell lines, established either by infection with virus isolated from feces or by HEV replicon transfection, its quantity is limited. This has been a major problem for developing an attenuated vaccine, which shall possibly work better than current subunit virus like particles with truncated capsid protein.

In our transcriptome analysis of HEV replicon transfected Huh7 cells, we observed a significant increase in long non-coding RNA (lncRNA) BISPR (BST2 interferon stimulated positive regulator) along with BST2 (Tetherin), which is positively regulated by BISPR [8]. This non-coding/coding gene pair forms part of the interferon induced innate immune system and are transcribed from a shared bidirectional promoter [9]. BST2 (Tetherin), which has been shown to antagonise the egress of several enveloped viruses, is expressed in most human tissues [10]. However, studies examining Tetherin expression in human livers by immunohistochemistry (IHC) are very few [10,11]; especially none in the setting of viral hepatitis. Our IHC studies showed distribution of BST2 (Tetherin) on bile canaliculi and an overexpression in HEV infected hepatocytes of acute liver failure patients. Further, co-localisation of Tetherin and HEV pORF2 was also observed in Huh7 cells *in vitro*.

This led us to functionally investigate the role of lncBISPR and BST2 (Tetherin) in egress of HEV. We generated BISPR gene deletion in Huh7 cells (ΔBISPR Huh7 cells) using CRISPR-Cas9 system and compared HEV egress from replicon transfected ΔBISPR Huh7 cells and wild type Huh7 cells. An eight fold increase in virus release was observed.

Further studies are needed to assess the *in vivo* effect of BISPR/BST2 in HEV induced hepatitis and to scale up the ΔBISPR Huh7 system to get enough *in vitro* egressed virus for studies on both infection as well as prevention.

## Materials and methods

Ethical clearance was obtained from the Institute Ethics committee (Approval number: IEC-49/09.12.2015), All India Institute of Medical Sciences, New Delhi, India.

### Cell culture, *in vitro* transcription and transfection

Huh-7 hepatoma cells [12] cultured in 1X DMEM (Life technologies, Carlsbad, California, United States), 10% FCS (Life technologies, Carlsbad, California, United States) and 1X Antibiotic antimycotic (Sigma Aldrich, St.Louis, Missouri, United States) at 37°C and 5% CO2 were used in all experiments. pSG HEV full length cDNA construct (FJ457024, genotype 1) was *in vitro* transcribed using mMessage mMachine IVT kit (Life technologies, Carlsbad, California, United States) as per the manufacturer’s instructions to produce ~7.2kb capped and poly-A tailed HEV replicon. Similarly, capped and poly-A tailed replication deficient HEV RNA was generated by *in vitro* transcription of pSGHEV-mut*RdRp* construct where GDD RdRp catalytic triad has been mutated to GAA [13]. Two micrograms of *in vitro* synthesised HEV RNA along with 50ng of pcDNA3-Fluc was transfected in 1.2 million Huh7 cells in T25cc culture flask (Corning, Sigma-Aldrich, United States) using Lipofectamine LTX reagent in serum and antibiotic free media as per guidelines (Life technologies, Carlsbad, California, United States). Four hours post transfection, media was replaced with complete media (1X DMEM with 10% FCS and 1X Antibiotic) and cultured for 12, 24 & 72 hours in separate flasks. Firefly luciferase activity was measured using Luciferase Assay system (Promega, Madison, Wisconsin, United States) and used to normalise the transfection efficiency.

### RNA Seq analysis of HEV transfected Huh7 cells using ion proton next generation sequencer

**Fig 1:**
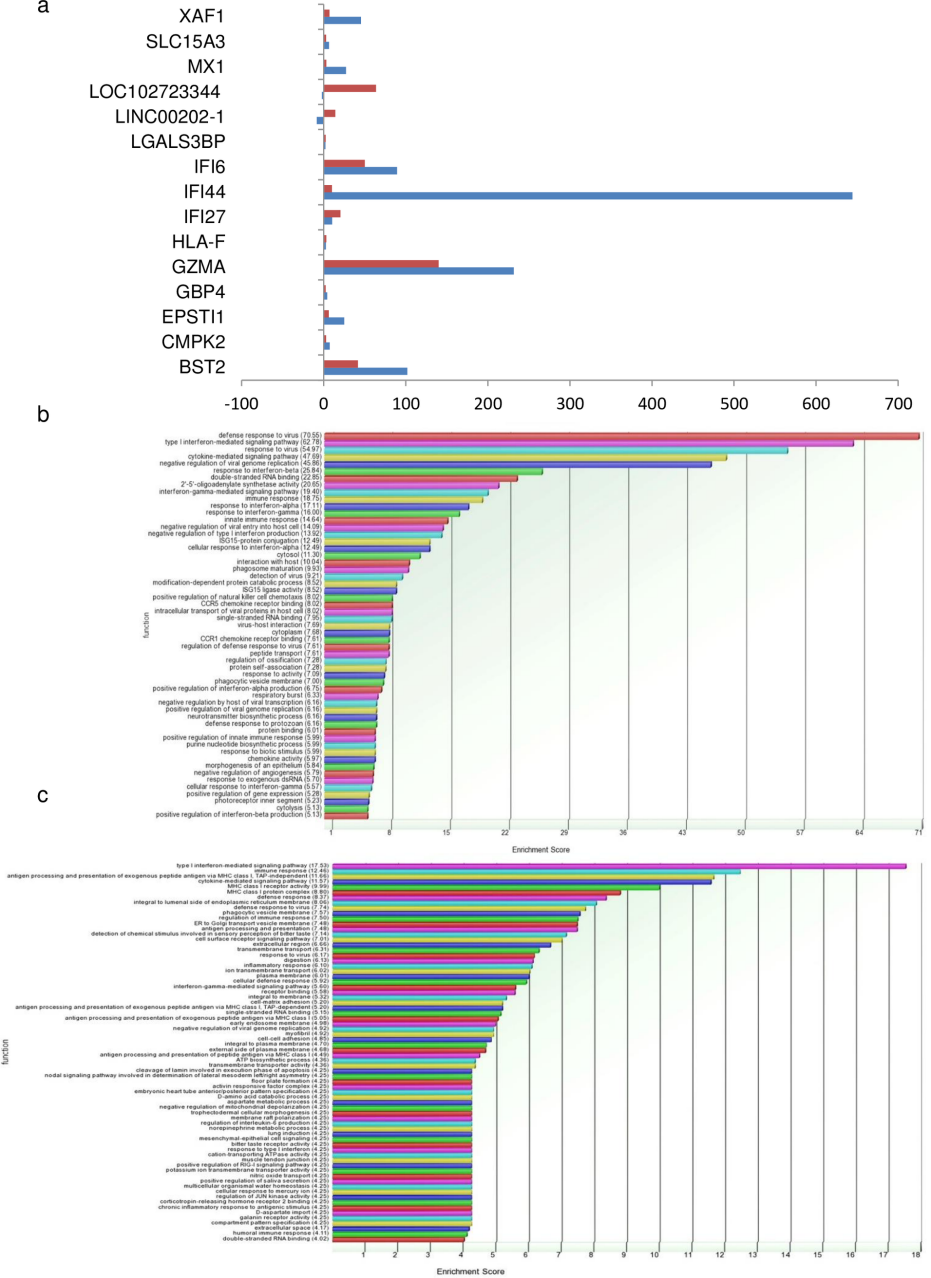
Graphical representation of RNA sequencing results. a. Bar chart representing fold changes of 15 mRNA/lncRNAs, differentially expressed in both 24 and 72 hr samples. Bars in blue and red represent fold change values of each gene at 24 and 72 hr respectively. b. Bar chart representing the major pathways observed to be enriched in Huh7 cells 24hrs post HEV transfection.c. Bar chart representing the major pathways observed to be enriched in Huh7 cells 72hrs post HEV transfection.

mRNA was purified from total RNA (isolated from control (n = 2) and HEV replicon transfected hepatoma cells at 24hrs (n = 2) and 72hrs (n = 2) post transfection) using Dynabeads mRNA Direct micro purification kit (Cat no: 61021, Life technologies, Carlsbad, California, United States) as per manufacturer’s instructions and quantitated using Qubit (Life technologies, Carlsbad, California, United States). Total RNA samples were spiked with appropriate quantity of ERCC Exfold controls as per given directions (ERCC, Cat no: 4456739 Life technologies, Carlsbad, California, United States) prior to mRNA isolation. 100 ng of ERCC spiked mRNA from each control and test sample was used for library preparation in separate reactions using Ion RNA Seq kit v2 (Cat no: 4475936, Life Technologies) as per manufacturer’s guidelines. Concentration and size distribution of all libraries was determined using DNA1000 chip (Agilent, Santa Clara, California, United States) on Bioanalyser 2100. Clonal amplification of cDNA libraries was performed by using Ion PI template OT2 Reagent 200 v3 (Cat no: 4488318, Life technologies, Carlsbad, California, United States). Template positive ISPs were recovered, enriched and processed for single-end forward sequencing on Ion Proton next generation sequencer using Ion PI chip and Ion PI sequencing reagents 200 v3 (Cat no: 4488315, Life technologies, Carlsbad, California, United States) as per manufacturer’s guidelines. Processing and analysis of RNA Seq data was done as previously described [14]. Briefly, output reads were trimmed, aligned and mapped using Star Aligner and Bowtie2 aligner in Partek flow software and differential expression analysis was done in Partek Genomic Suite version 6.6. Differential expression analysis was performed on RPKM normalised and log transformed read counts by analysis of variance (ANOVA). Genes with fold change > = 2 and p-value < 0.05 were considered for differential expression and gene ontology analysis (Fig 1).

### Confirmation of BISPR/BST2 (Tetherin) upregulation pattern

**Fig 2:**
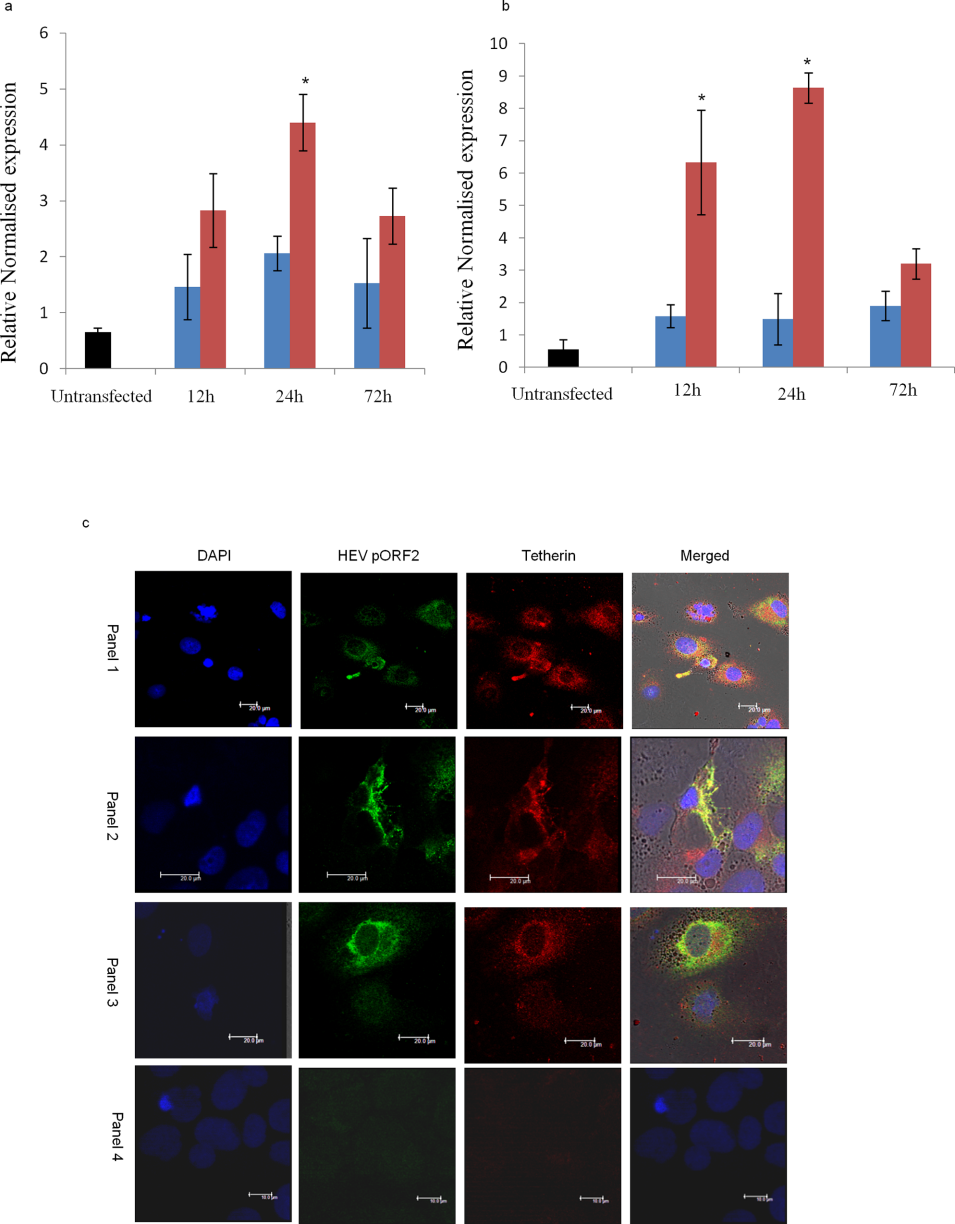
BST2 (Tetherin) and BISPR expression in HEV transfected Huh7 cells. a. Bar chart representing BST2 (Tetherin) RNA levels in untransfected (black), wild type HEV replicon (red) and replication deficient HEV RNA (blue) transfected Huh7 cells at 12, 24 and 72 hrs post transfection. Relative expression values have been normalised with respect to GAPDH gene (n = 3; error bars represent standard deviation, *p value <0.01). b. Bar chart representing BISPR RNA levels in untransfected (black), wild type HEV replicon (red) and replication deficient HEV RNA (blue) transfected Huh7 cells at 12, 24 and 72 hrs post transfection. Relative expression values have been normalised with respect to GAPDH gene (n = 3; error bars represent standard deviation, *p value <0.01). c. Confocal images (40x objective) representing dual Immunofluorescence colocalisation patterns of HEV pORF2 and BST2 (Tetherin) in HEV transfected Huh7 cells in replicate samples (panels 1, 2: cytoplasmic and membranous, 3: perinuclear), 24hrs post transfection. Panels 4 represents staining pattern of untransfected Huh7 cells. Bars in panels 1–3 and 4 represent 20μm and 10μm respectively.

Whole transcriptome RNA-Seq analysis performed in HEV replicon transfected Huh7 cells at 24hrs and 72hrs post transfection (Sequence Read Archive, BioProject ID PRJNA381374) showed an increase in the expression of both BISPR (147 folds) and BST2 (Tetherin) (101 folds) at 24hrs. At 72hrs, however, only BST2 (Tetherin) was upregulated (40 folds). We therefore sought to validate this temporal expression pattern observed in lncBISPR and BST2 (Tetherin) genes by qPCR (Fig 2A and 2B).

Twenty four & 72hrs post transfection, total RNA was isolated from cultured/transfected Huh7 cells using Trizol reagent (Life technologies, Carlsbad, California, United States) and converted to cDNA using 1pmol oligo dT/gene specific primer in 20μl reaction using AccuScript High Fidelity 1^st^ strand cDNA synthesis kit (Agilent, Santa Clara, California, United States). Two microliters of this cDNA was used in 20μl real time reaction mixture containing 1pmole each of gene specific forward and reverse primer and 1X Ssofast Evagreen supermix (Bio-Rad, Hercules, California, United States). Q-PCR amplification was performed on CFX96 real time PCR machine (Bio-Rad, Hercules, California, United States) as follows: 95°C for 2min, 40 cycles of 95°C for 10 sec, 55°C for 30 sec (combined annealing/extension step) followed by melt curve analysis from 65°C to 95°C with 0.5°C increments. Differential gene expression analysis was done on BIORAD CFX manager software using GAPDH as housekeeping control for normalisation. S1 Table shows the sequence of BISPR and BST2 (Tetherin) primers used in real time PCR.

### Immunofluorescence assay for BST2 (Tetherin) and HEV capsid protein (pORF2) in HEV infected Huh7 cells

Dual Immunofluorescence staining of HEV transfected Huh7 cells was performed using mice anti-BST2 (10μg/ml, Cat no: ab88523, Abcam, Cambridge, United Kingdom) and in-house Rabbit polyclonal anti-pORF2 antibody (1:1200) following the protocol previously described [14]. 1:1200 dilution of Goat anti-mice Alexa 647 plus (Cat no: A32728, Molecular Probes, Eugene, Oregon, United States) and Donkey anti-Rabbit Alexa 555 (Cat no: ab150062, Abcam, Cambridge, United Kingdom) secondary antibodies were used. Stained cells were mounted using ProLong® Diamond antifade mountant with DAPI (Cat no: P36971, Life Technologies, Carlsbad, California, United States) and observed in sequential scan mode with 405nm, 561nm and 633nm laser lines (Fig 2C) using dual Hybrid detector of LeicaTCS-SP5 confocal microscope (Leica, Wetzlar, Germany).

### Immunohistochemistry for BST2 (Tetherin) and HEV pORF2 expression in liver biopsy specimens

**Fig 3:**
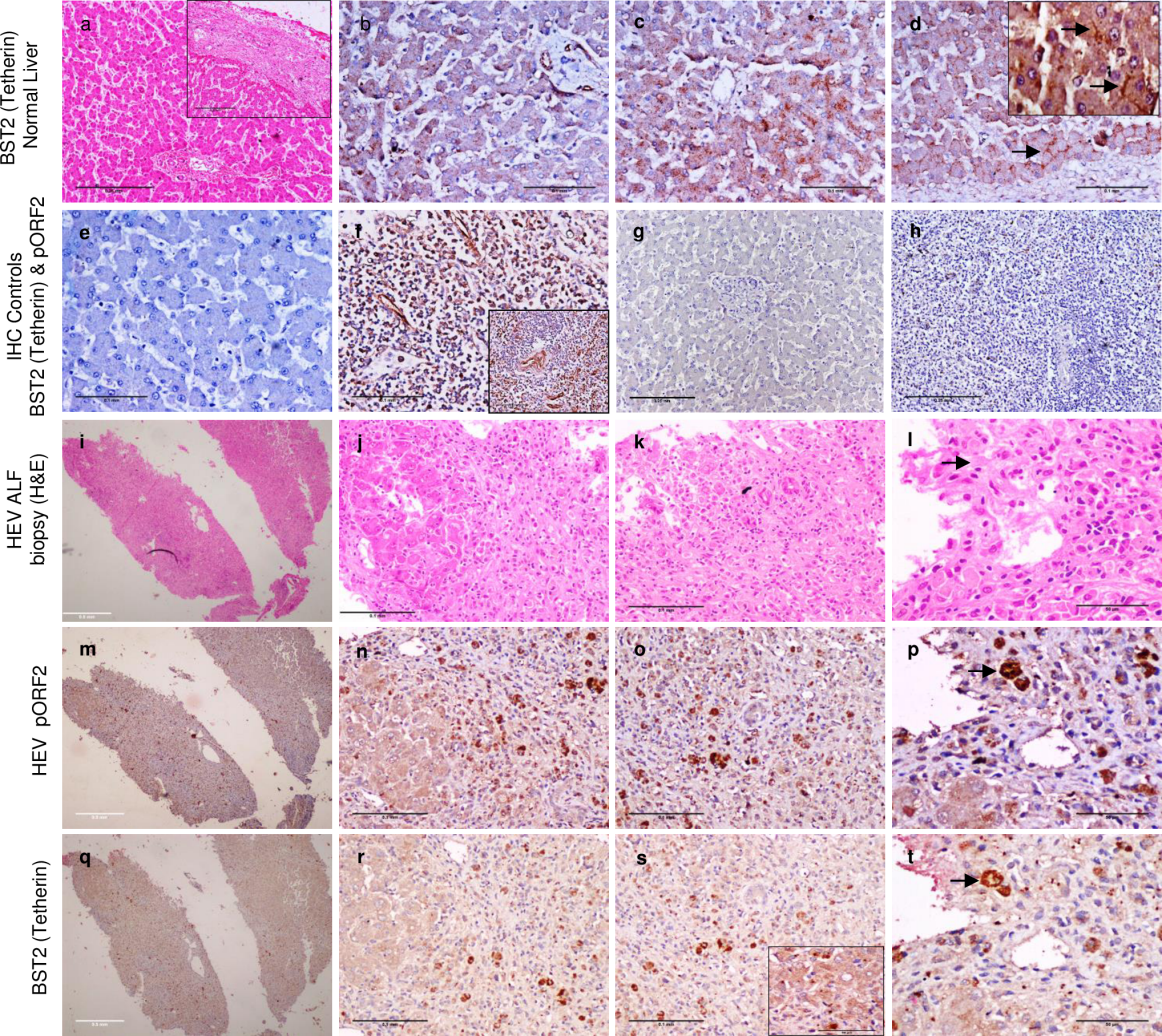
Representative images showing immunohistochemical expression of BST2 (Tetherin) and HEV pORF2 *in vivo*. Expression of BST2 (Tetherin) in histologically normal liver [a-d (inset in ‘a’ represents subcapsular region)], lymph node (f) and spleen (inset f). Blood vessels in liver (b) served as positive internal control for BST2 (Tetherin) staining. Hepatocytes show cytoplasmic (c) and canalicular staining (arrows in Fig d and inset Fig d). Figs ‘e’ (liver) and ‘h’ (spleen) represent negative IHC controls with secondary antibody only. Fig ‘g’ represents negative control for pORF2 staining in histologically normal liver. Figs ‘i-t’ depict immediate serial sections of HEV associated Acute liver failure tissue biopsy (ALF) stained with Hematoxylin and Eosin (H&E; i-l), anti-HEV pORF2 IHC (m-p) and anti-BST2 (Tetherin) IHC (q-t). Similar cell populations stained with both the antibodies (Arrows in Figs l, p and t). Inset in Fig ‘s’ depicts perinuclear staining of BST2 (Tetherin) in HEV associated ALF case. Images ‘i, m, q’ were taken with 4x objective (Bar = 0.5mm); ‘a, h, g’ with 10x objective (Bar = 0.25mm); ‘b, c, d, e, f, g, j, k, n, o, r, s’ with 20x objective (Bar = 0.1mm) and ‘l, p, t, inset d and inset s’ with 40x objective (Bar = 50μm).

*In vivo* expression levels of BST2 (Tetherin) were studied in HEV associated acute liver failure biopsies (n = 2) retrieved from archives of Department of Pathology, AIIMS, New-Delhi. Normal liver for control (n = 4) was obtained from autopsies performed for non-hepatic diseases (Pancreatitis) or wedge biopsies of liver carried out during other surgical procedures (lino-renal shunt, splenectomy, esophagectomy). Ethical clearance for use of human tissues was obtained from institutional ethical clearance committee (IEC-49/09.12.2015). Immunohistochemistry (IHC) was performed on 4μm thin formalin-fixed paraffin embedded tissue sections post heat induced epitope retrieval (10 mM Citrate buffer, pH 6.0) using 1:600 dilution of mice anti-BST2 (Cat no: ab88523, Abcam, Cambridge, United Kingdom) and 1:50 dilution of in-house monoclonal mice anti-HEV pORF2 primary antibodies as previously described [15]. Splenic and lymph node tissues were used as positive controls for BST2 immunostaining. BST2 (Tetherin) and HEV pORF2 staining was done on immediate serial sections to determine co-expression in similar cell populations (Fig 3I–3T). Images were taken using Nikon ECLIPSE E600 and Digital Sight DS-5M-L1 (Nikon, Minato, Tokyo, Japan).

### CRISPR-Cas9 mediated generation of ΔBISPR Huh7 cells

#### Cloning of dual guide RNAs in Cas9 vector and homology arms in HR donor vector

**Fig 4:**
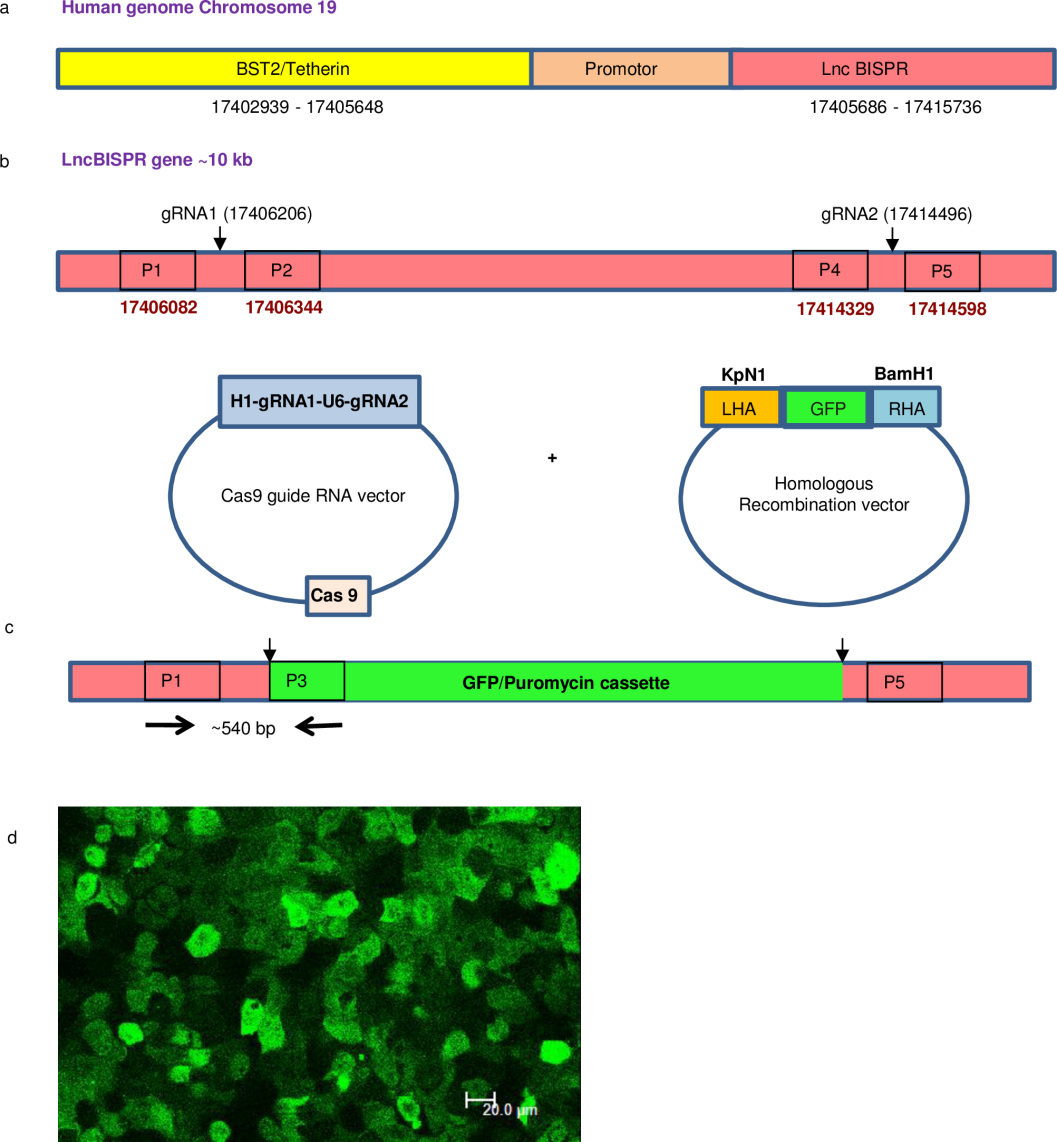
Design of CRISPR-Cas9 mediated lncBISPR gene deletion using dual gRNA-Cas9 and homologous recombination (HR) donor constructs. a. Genomic organisation of BST2 (Tetherin) and LncBISPR gene on human chromosome 19. b. Schematic representing the targeting sites of gRNA1 and gRNA2 in exon 2 and exon 5 of lncBISPR gene respectively. c. Schematic representing the location of primers P1 and P3 used for PCR screening of BISPR gene deletion. d. GFP positive Huh7 cells, post dual gRNA-Cas9, HR donor construct transfection and Puromycin selection (40x objective, Bar = 20μm).

Guide RNAs targeting human LncBISPR gene (chromosome 19, 17405686–17415736, Ncbi Accession no: NC_000019.10) were designed using CRISPR design tool (crispr.mit.edu; Fig 4A). Guide RNAs at locus 17406206 in exon 2 (score: 88) and 17414496 in exon 5 (score: 93) had a high score for specific cleavage and minimum off-site targets. Both gRNAs (S1 Table) and U6 block (Systems Biosciences, Palo Alto, California, United States) were used as template to perform fusion PCR and generate ~450 bp H1-gRNA1-U6-gRNA2 amplicon (S1 Fig) using Precision X^TM^ Cas9 SmartNuclease system (CAS740A). This amplicon was then ligated into linearized Precision-X Cas9 vector as per manufacturer’s instructions (Precision X^TM^ Cas9 SmartNuclease system, Systems Biosciences, Palo Alto, California, United States). Final screening of colonies carrying dual gRNA ligated Cas9 constructs was done by Sanger sequencing using H1 primer.

The left and right homology arms to be cloned at *KpN1* and *BamH1* sites of HR vector (Cat no: HR410PA-1, Systems Biosciences, Palo Alto, California, United States) were PCR amplified from region 17405497–17406190 and 17414509–17415316 (on chromosome 19) respectively from Huh7 genomic DNA using combination of Taq (Applied Biosystems, Foster City, California, United States) and *Pfu* DNA polymerase (Promega, Madison, Wisconsin, United States) in a 4:1 ratio, gel eluted, digested with *KpN1* and *BamH1* respectively and sequentially ligated into HRP410 vector using T4 DNA Ligase (NEB, Ipswich, Massachusetts, United Kingdom). Sequence of primers used for amplification of homology arms is given in S1 Table. Sequence and orientation of both homology arms in the positive construct were verified using Sanger sequencing.

#### Transfection of Cas9-gRNA and HR donor constructs and selection of ΔBISPR Huh7 cells by fluorescence activated cell sorting

Two micrograms each of gRNA-Cas9 and HR donor construct were transfected in 1.2 million Huh7 cells as described above. Seventy two hours post transfection cells were selected in presence of 5μg/ml Puromycin for 10 days. High GFP positive Huh7 cells were sorted twice (14 and 21 days post transfection) from culture of puromycin resistant GFP positive Huh7 cells using FACS Aria III instrument (Becton Dickinson, New Jersey, United States) and BD FACS Diva ver6.1.3 software (S2 Fig). These cells were seeded in 96 well plates (one cell per well) by cell sorter as well as by limiting dilution and propagated to obtain monoclonal colonies. Eight colonies were stable which could be cultured to T25 culture flasks and were screened for deletion of lncBISPR gene.

#### Validation of lncBISPR deletion in Huh7 cells using PCR, sanger sequencing, qPCR and immunofluorescence

Genomic DNA was isolated from one million cells from each of the eight colonies and screened by PCR using primers flanking different regions of genomic DNA and Puromycin/GFP cassette (from HR vector). Fig 4B and 4C depict the locations of primers used to screen for deletion of LncBISPR gene and insertion of Puromycin/GFP cassette in Huh7 cells. P1, P2, P4 and P5 (S1 Table; Fig 4B) represent the primers specific to lncBISPR gene in human genomic DNA. P3 represents the primer specific to Puromycin/GFP cassette of HR donor vector (Fig 4C). DNA isolated from Huh7 colonies was screened using PCR with P1, P3 and P1, P2 primers to identify colonies with successful insertion of Puromycin/GFP cassette (MCS1-EF1a-GFP-T2A-Puro-pA-MCS2). This HR cassette is flanked by insulator sequences at both the ends (Cat no: HR410PA-1, Systems Biosciences, Palo Alto, California, United States). A ~540 bp product with P1 and P3 primers indicated insertion of HR cassette at the targeted site (S1 Fig). A specific 540 bp band was observed in 2 of the 8 colonies screened. Genomic DNA from both these colonies did not show any PCR amplification with P1 and P2 primers indicating homozygous deletion (at both the alleles) of BISPR gene in them. Cells from one of these 2 colonies grew well and experiments were performed in these cells (ΔBISPR Huh7 cells).

Genomic DNA sample from this colony was also screened using primers specific to HR donor vector (BamH1 HR forward and P6 reverse) to rule out the possibility of persistence of HR donor vector. According to our primer design strategy, a ~1000 bp product indicates the presence of HR donor vector. A 1000 bp band was observed in positive control (i.e. Purified HR donor plasmid with both homology arms cloned) but not in the genomic DNA extracted from positive colony (S1 Fig) indicating successful insertion of HR GFP/Puromycin cassette and absence of free/integrated HR donor vector in those cells. Further confirmation of BISPR gene knock out was done using Sanger sequencing of 540bp PCR amplicon with P1 primer.

**Fig 5:**
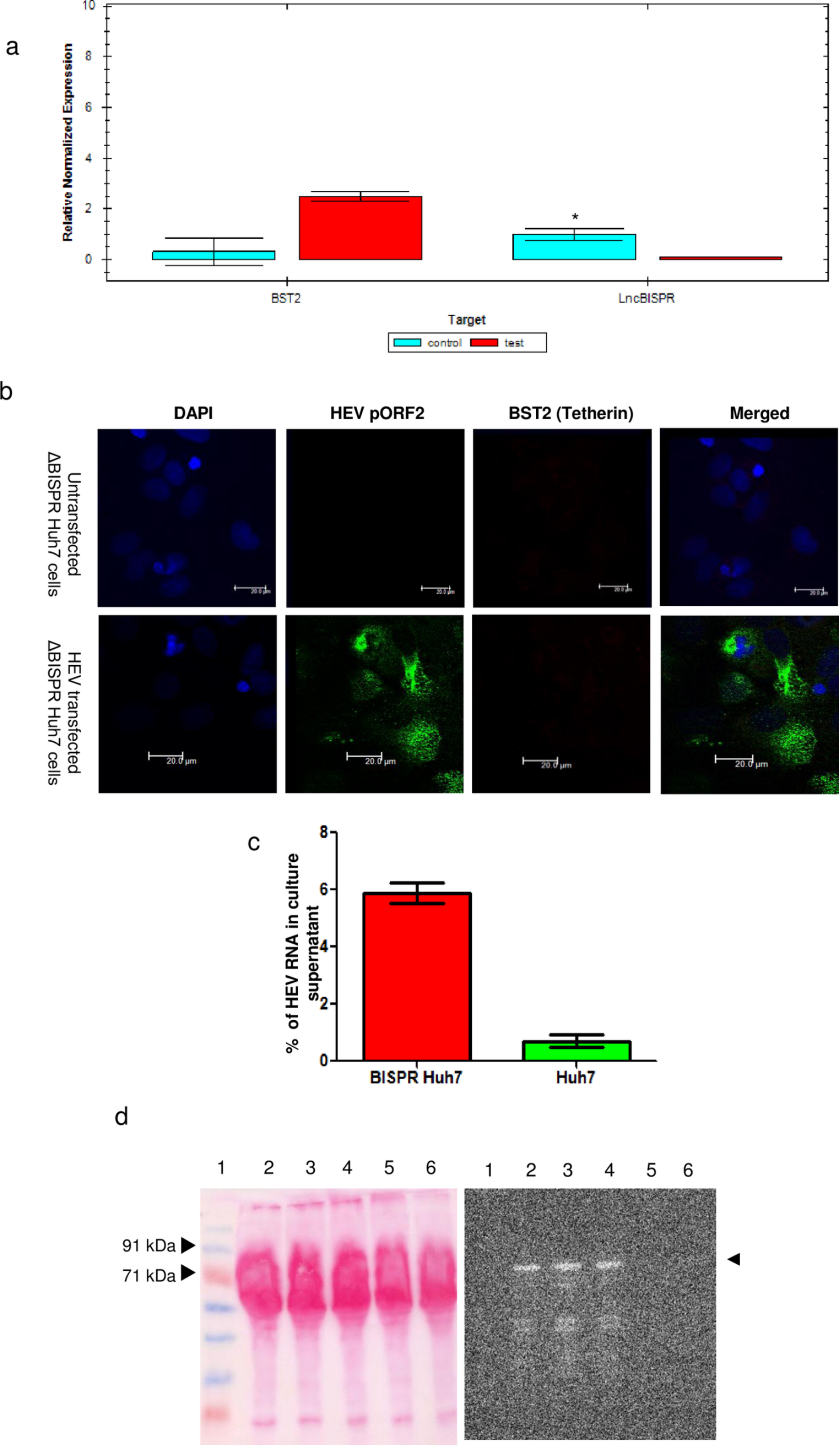
Analysis of HEV virion egress from ΔBISPR Huh7 cells. a. Bar diagram showing expression of lncBISPR and BST2 (Tetherin) in HEV replicon transfected ΔBISPR Huh7 (test) 24 hrs post transfection and untransfected Huh7 cells (control) (n = 3; error bars represent standard deviation, *p-value <0.01). GAPDH was used as a reference gene for normalization. b. Confocal images (40x objective) representing staining patterns of HEV pORF2 and BST2 (Tetherin) in untransfected and HEV transfected ΔBISPR Huh7 cells, 24hrs post transfection. Bars represent 20μm. c. Bar diagram representing percentage of HEV RNA in culture supernatants of HEV transfected ΔBISPR Huh7 and Huh7 cells at 24 hrs post transfection (n = 3; error bars represent standard deviation). d. Ponceau-S and anti-HEV pORF2 stained western blot analysis of HEV virions in the culture supernatants of HEV transfected Huh7 (lane 6) and ΔBISPR Huh7 cells (lanes 2–4) 24hrs post transfection. Capsid protein pORF2 is shown by arrow heads. Lane 5 represents the negative control sample (culture supernatant from untransfected Huh7 cells). Lane 1 represents prestained protein molecular weight marker (Puregene, United States).

Functional validation of BISPR deletion was done by qPCR quantitation of BISPR and BST2 RNAs (Fig 5A) and BST2 immunofluorescence staining (Fig 5B) in HEV replicon transfected ΔBISPR Huh7 cells. No BISPR RNA could be detected in ΔBISPR Huh7 cells 24hrs post HEV transfection confirming BISPR gene deletion in them. Similarly, no upregulation in BST2 expression could be detected in HEV transfected ΔBISPR Huh7 cells upon immunofluorescent staining with anti-BST2 (Tetherin) and anti-pORF2 antibodies (Fig 5B).

### Virion release assay using real time PCR

In vitro transcribed HEV replicon was transfected in both native Huh7 and ΔBISPR Huh7 cells (1.2 million each) and cultured for 24 and 72hrs post transfection in separate flasks, as described above. Culture media from these cells was removed at 24 and 72 hrs, centrifuged at 1200 rpm for 40 minutes at 4°C and passed through a 40μm filter. These culture supernatants were treated with 2U of RNAse A (Life Technologies, Carlsbad, California, United States) at 37°C for 2hrs to degrade any free RNA molecules present in the supernatant. A RNA spike in control was included to assess complete degradation of RNA. Post RNase A treatment, virus encapsidated RNA was isolated from 100μl of culture supernatant using Trizol reagent and stored at -80°C. Adherent cells in the flasks were washed twice with 1x PBS and total cellular RNA was isolated using Trizol reagent and stored at -80°C.

Equal volume of RNA isolated from each cellular lysate and supernatant was used for first strand cDNA synthesis using HEV specific reverse primer (HEV Rev, S1 Table) and AccuScript High Fidelity 1^st^ strand cDNA synthesis kit (Agilent, Santa Clara, California, United States) as per instructions. Quantitative real time PCR was performed with 5μl cDNA with HEV For and Rev primers (S1 Table) using reaction composition and conditions described above. Standard curve for absolute copy number quantification was generated with serial 100 fold dilutions (from 10^8^ to 10^2^) of cDNA made from in vitro transcribed HEV RNA. Percentage of virus release in each case was calculated as: {Virus Copy no. in culture supernatant/Total virus copy no. (lysate + culture supernatant) x 100} (Fig 5C).

### Western blot analysis of HEV pORF2 in culture supernatant

Culture supernatant from HEV transfected ΔBISPR Huh7 cells (24h post transfection) was pooled and concentrated using Pierce protein concentrators (Thermo Scientific, Waltham, Massachusetts, United States). 50μl of this concentrated culture supernatant was dissolved in 6x SDS Page loading buffer, separated by SDS PAGE on 10% polyacrylamide gel and transferred to 0.45 mm PVDF membrane (Thermoscientific, Waltham, Massachusetts, United States) as previously described [16]. Equal volume of pooled and concentrated culture supernatant from untransfected (negative control) and HEV transfected Huh7 cells was processed similarly. Normalisation of protein load for all samples (proteins secreted in culture media) was done by Ponceue-S staining of western blots before BSA blocking. The membrane was stained using in-house rabbit anti-pORF2 polyclonal primary antibody, goat anti rabbit HRP conjugated secondary antibody (Cat no: P0448, Dako, Glostrup Municipality, Denmark) and developed using SuperSignal west pico chemiluminescent substrate (Cat no: 34077, Thermo Scientific, Waltham, Massachusetts, United States) (Fig 5D).

## Results

### RNA Seq analysis of HEV transfected Huh7 cells

A total of 148 and 121 mRNAs were found to be differentially expressed in 24hr and 72hr samples respectively with 15 genes being common to both time points (Fig 1A). Similar to our previous study [14], a high representation of interferon and immune related genes was noted. While the upregulated target mRNAs represented genes of diverse pathways such as interferon (IFIT5, IRF9, IFI44, IFI27, IFI6), chemokines (CCl4, CCL5, CXCL10, CXCL9), apoptosis (BIRC3), and anti-viral responses (TRIM22). Downregulated target mRNAs represented cell surface molecules (HS6ST3, DENND1C). Amongst the long non-coding RNAs, BISPR, LINC00202-1 and LOC102723344 showed highly significant differential alterations. Differentially expressed mRNAs and lncRNAs have been listed in S3 Fig.

Even in the 15 genes, DE at both 24hrs and 72hrs, 4 of the 5 interferon inducible genes (BST2, MX1, IFI44 and IFI6) show many fold higher expression at 24hrs compared to 72hrs (Fig 1A & S3 Fig); in line with the global reduction in interferon response observed by 72hrs.

The function of differentially expressed (DE) mRNA genes has been studied by both Gene ontology tool of Partek Genomic Suite (version 6.6) and by literature search. Most of the DE genes 24hrs post HEV transfection belonged to host defense response to virus, Type I interferon mediated signalling pathway, cytokine mediated signalling pathway, interferon gamma mediated signalling pathway, negative replication of viral genome replication, negative regulation of viral entry into host cells etc. At 72 hrs, we observed reduction in host immune response with lowered enrichment score for the above pathways. Fig 1B and 1C show bar charts representing enriched pathways in HEV transfected Huh7 cells 24 and 72hrs post HEV replicon transfection respectively.

### Temporal co-expression pattern of lncBISPR/BST2 (Tetherin) in HEV infection in-vitro

In our RNASeq analysis, we focussed on mRNA and lncRNA pairs which are known/ predicted to have functional correlation and showed significant differential expression. We observed that while at 24hrs both Lnc BISPR (147 folds, p<0.01) and BST2 (Tetherin) (101 folds, p<0.01) were highly upregulated; at 72 hrs, BST2 (Tetherin) expression was comparatively reduced (40 folds, p<0.01), accompanied by normal levels of BISPR. These alterations may be an indirect fall out of overall reduction in the interferon and inflammatory activity reported during HEV infection [17,18]. Corroborative alterations in support of this were also observed in our RNASeq data in the form of reduced representation at 72hrs of several chemokines and Interferon stimulated genes which were highly upregulated at 24hrs post replicon transfection. lncBISPR is known to be interferon induced and a part of innate immune response playing a role in virus egress through BST2 (Tetherin).

Alterations in the expression of BST2 (Tetherin) and lncBISPR at the two time points were further confirmed using gene specific primers in Real time PCR (Fig 2A and 2B). Levels of BST2 and BISPR RNA in replication deficient HEV RNA transfected cells were found to be less than ~2 folds at all the time points studied (12, 24 and 72 hrs) indicating the magnitude of induction of immune responses upon foreign RNA transfection. However, in Huh7 cells transfected with replicating HEV replicon BST2, BISPR RNAs were found to be ~2.8, ~6.2 and ~4.2, ~8.6 folds at 12 and 24 hrs post transfection respectively. Similar to the observations of our transcriptome data, BST2, BISPR RNA (~2.6, ~3.1) upregulation was found to comparatively reduce by 72hrs. These findings suggest functional corroboration between BST2/BISPR RNA upregulation and HEV replication. This reveals a pattern of temporal alterations of BISPR and BST2 (Tetherin) in HEV infected hepatoma cells in line with HEV replication cycle described earlier [13]. These alterations may however vary between cell lines due to differences in transfection efficiency, magnitude of host innate response and replication efficiency of HEV which are well known to differ in various cell lines [19].

### BST2 (Tetherin) colocalizes with HEV capsid protein in vitro

To visualise the expression and localisation of both BST2 (Tetherin) and HEV capsid protein (pORF2) in HEV infected hepatocytes, we performed dual immunofluorescence staining of full length HEV replicon transfected Huh7 cells with anti-BST2 and anti-pORF2 antibodies. BST2 (Tetherin) was found to be upregulated in HEV transfected cells compared to untransfected Huh7 cells (Fig 2C). Membranous, cytoplasmic and perinuclear co-localisation of BST2 (Tetherin) and HEV pORF2 was observed (Fig 2C). An average of ~40% of hepatoma cells showed co-staining for HEV pORF2 and BST2 (Tetherin).

### BST2 (Tetherin) is upregulated in HEV infected hepatocytes *in vivo*

Anti-BST2 (Tetherin) immunohistochemistry on histologically normal liver tissues (Fig 3A–3D) revealed a dominant canalicular staining pattern (Fig 3D and 3D inset; arrows) along with granular cytoplasmic (Fig 3C) positivity of variable intensity in different hepatocytes. Hepatic artery branches in the portal triad served as internal positive controls (Fig 3B). No staining was observed in bile ducts.

In HEV associated acute liver failure (Fig 3I–3T), there was an overall increase in the intensity of anti-BST2 (Tetherin) staining in hepatocytes. This was however distributed diffusely in the cytoplasm (Fig 3R–3T) and was so intense that localisation to domains of hepatocytes could not be ascertained in most cells. Few hepatocytes showed a perinuclear staining pattern (Fig 3S, inset). IHC with anti HEV pORF2 on serial sections revealed a similar pattern of staining in same populations of hepatocytes (Fig 3M–3P). Further, foci of hepatocytes which did not stain with anti-pORF2 were also found to be only weakly BST2 (Tetherin) positive (Fig 3N, 3R, 3P and 3T). Canalicular staining pattern of BST2 (Tetherin) in HEV ALF could not be delineated. Our findings indicate that BST2 (Tetherin) upregulation in response to HEV infection occurs *in-vivo* as well. An average of ~40% of hepatocytes showed co-staining for HEV pORF2 and BST2 (Tetherin) proteins in vivo as well.

### HEV virion egress from ΔBISPR Huh7 cells

We performed real time PCR to quantify copy numbers of HEV RNA (positive strand) in both culture supernatants and cell lysates of HEV transfected ΔBISPR Huh7 and Huh7 cells at 24 and 72hrs post transfection. The exact copies of HEV RNA detected in cell lysate and supernatant of HEV transfected ΔBISPR Huh7 and Huh7 cells (at both time points) have been listed in S2 Table. Percentage of HEV RNA released was calculated as described above. We observed ~8 fold increase in HEV positive strand RNA in culture supernatant of ΔBISPR Huh7 cells at 24hrs (Fig 5C). However at 72hrs the difference between virus egress from **Δ**BISPR and naive Huh7 cells was not significant. This is expected, as the interferon induced BST2 response in HEV transfected Huh7 cells is also reduced by 72hrs, as observed in transcriptome data. **I**ncreased levels of HEV positive strand RNA in culture supernatants of HEV infected ΔBISPR Huh7 cells indicate the regulatory role of BISPR/BST2 gene pair in HEV life cycle, possibly its egress.

At 24 hrs, confirmation of HEV virions in culture supernatant of HEV transfected ΔBISPR Huh7 cells was done by probing for HEV capsid protein pORF2 by Western blot (Fig 5D). The molecular weight of capsid protein of HEV virions is observed to be higher than ~72 kDa which could possibly be due to the glycosylation of pORF2 capsid protein as previously reported on the surface of HEV virions secreted in cell culture models [20].

## Discussion

Hepatitis E virus (HEV) mostly spreads through feco-oral route. The virus exists [1] in two forms; as an enveloped virus in blood and as unenveloped virus in bile. Our earlier studies have shown that the capsid protein in virus from bile is truncated [21]. There is a possibility that this truncation occurs either before or after the egress of HEV from liver cells [1]. Several proteases have been presumed to be involved in this process: the endogenous protease of the virus; the rhomboid membrane protease which gets upregulated by the pORF3 protein of virus; or a totally novel protease unknown till now. These varying concepts make study of virus egress in case of HEV interesting. It is known that the capsid protein (pORF2) is present in both glycosylated and non-glycosylated forms [22,23] and undergoes membrane translocation on endoplasmic reticulum [6]. Virus replication occurs on the endoplasmic reticulum (ER) membrane [5]. Therefore most probably the virus assembly occurs on the ER membrane.

HEV replication and life cycle is well studied and reported in Hepatoma cell lines (Huh7 and HepG2) by several groups [13,19,24–26]. The kinetics of HEV replication cycle in culture system used in our present study [i.e. Huh7 cells and in vitro transcribed full length HEV replicon with 5’ cap and 3’ poly A tail] are well studied and documented [13]. In similar culture system, we have earlier reported the presence of HEV negative strand as early as 4hrs post HEV replicon (FJ457024) transfection [13]. We had also reported that peaks corresponding to HEV subgenomic RNA (which forms HEV pORF2 and pORF3) were observed at 8, 14 and 24 hrs post transfection. Also, the expression of HEV RNA dependent RNA polymerase (RdRp) has been documented to be maximum at 24 hrs post HEV replicon transfection in HepG2 cells [24].

In present study, our transcriptome analysis of HEV transfected Huh7 cells showed increased expression of paired noncoding/coding RNAs of BISPR/BST2 (Tetherin) 24 and 72hrs post transfection (Sequence Read Archive, BioProject ID PRJNA381374). Tetherin has been implicated in egress of several other enveloped viruses such as HIV, Ebola virus, HCV and HBV [27–29]. Therefore we investigated the egress of HEV in ΔBISPR Huh7 cells in comparison to naïve Huh7 cells. We made four important observations: 1) In human liver, Tetherin has a membrane distribution with a particular affinity to bile canalicular membrane (Fig 3D) in addition to the cytoplasmic localisation (Fig 3C) reported in literature [10]; 2) In human liver failure due to HEV, there is an overexpression of Tetherin in the HEV infected hepatocytes (Fig 3R–3T). In this regard, we note that the canalicular Tetherin staining observed in histologically normal liver could not be clearly delineated in HEV ALF cases due to a loss of the lobular architecture coupled with diffuse intense cytoplasmic Tetherin positivity in HEV infected hepatocytes. It is possible that such a pattern is preserved in the earlier stages of HEV hepatitis. 3) Tetherin and capsid protein (pORF2) co-localise in cytoplasm and cell membrane (Fig 2C) and 4) In tissue culture, the egress of virus as measured by qPCR of HEV RNA and Western blot of capsid protein pORF2, is increased by eight folds in ΔBISPR Huh7 cells. Therefore it is fair to believe that BISPR and BST2 (Tetherin) play an important role in HEV egress.

These findings throw up particularly interesting possibilities in case of HEV. Coupled with the reports of the normal intracellular transport and recycling of Tetherin molecules [30], its location and function in organizing lipid rafts on the cell membrane [31], its clathrin dependent endocytosis [32], its linkage to actin cytoskeleton and studies documenting an important role of Tetherin in actin reorganisation in polarized epithelial cells [33]; these players are all too familiar in the landscape of HEV replication and egress. HEV replicates in Endoplasmic reticulum and has to migrate towards the bile canalicular surface of the hepatocytes for egress into bile and for further transmission to a susceptible host. Egress into blood is largely a dead end as far as natural virus propagation is concerned. How does HEV manage this? HEV pORF3 in association with hepatocyte cytoskeleton [4] and cellular ESCRT (endosomal sorting complexes required for transport) machinery [34] are thought to be chief contributors. Given the information available about BST2 (Tetherin), along with our finding of its canalicular expression, it is quite possible that Tetherin also contributes to the polarized transport and egress of HEV virions.

In such a scenario, overexpression of Tetherin would be, counter-intuitively, beneficial to HEV as more virions could reach the canalicular surface and once exposed to canalicular bile contents, lose the envelop and shed into bile without getting endocytosed and digested as happens in other viruses [35]. Host proteases expressed on hepatocyte membrane may also aid in this process of “untethering”. If this is true, then HEV evolution should select against development of viral BST-2 antagonists which are found in many viruses [36] particularly the ones which disrupt trafficking of BST2 (Tetherin) to the cell surface [37]. This remains to be tested.

The finding of increased virus egress from ΔBISPR Huh7 cells has a practical application too. The possibility of developing an attenuated vaccine for HEV has mostly been unattainable due to low level of virus in culture supernatant obtained either from replicon transfected cells or by infection with HEV virions isolated from feces. If BISPR deletion increases the amount of virus released into culture supernatant, then attempts can be made to provide sufficient virus to produce inactivated HEV virus vaccine.

## Supporting information

S1 Fig Generation of dual gRNA-Cas9, homologous recombination (HR) donor constructs and validation of ΔBISPR Huh7 cells. a. 2% agarose gel image representing ~450 bp H1-gRNA1-U6-gRNA2 amplicon generated by fusion PCR (lanes 2 and 3). Lane 1 represents 50bp DNA ladder (BR Biochem, New-Delhi, India).b. 1.2% agarose gel image showing analysis of DNA fragments after *EcoRI* and *NdeI* restriction digestion of HR donor vector (with both left and right homology arms ligated in correct orientation). DNA fall-out of ~2.6kb indicates positive constructs (lanes 2–5). Lane 1 shows 1kb DNA ladder (BR Biochem, New-Delhi, India).c. 2% agarose gel image representing PCR based screening of genomic DNA isolated from eight single cell colonies (Huh7) with primers P1and P3 to identify BISPR deletion. Lane 1 represents no template control. Lane 4 and 6 represent colonies positive for BISPR deletion showing ~540 bp amplicon. Lanes marked M represent 50bp DNA ladder (BR Biochem, New-Delhi, India).d. 1.5% agarose gel representing PCR amplification of genomic DNA isolated from eight single cell colonies (Huh7) with HR vector specific primers (BamH1 HR forward and P6 reverse). Lanes 1 and 5 represent no template controls. Lanes 2 and 6 show ~1000bp amplicon from HR donor vector (positive control). No such amplification could be detected in genomic DNA isolated from ΔBISPR Huh7 cells (lanes 3 and 7) and Huh7 cells (lanes 4 and 8). Lane M shows 1kb DNA ladder (BR Biochem, New-Delhi, India).
(PPT)

S2 Fig Fluorescence activated cell sorting of high GFP positive Huh7 cells after puromycin selection. a. Profile of control Huh7 cells using blue laser in GFP channel.b. Profile of puromycin resistant Huh7 cells, sorted 14 days post Cas9- gRNA and HR donor vector transfection for high GFP expressing cells.c. Profile of second sort of puromycin resistant Huh7 cells, performed 21days post transfection (7 days after first sort).d. Post sort profile of cells after second sorting (21days post transfection).
(PPT)

S3 Fig List of mRNAs and lncRNAs differentially expressed in RNA seq analysis of HEV transfected Huh7 cells. (XLS)

S1 Table Sequence of primers used in the study. (PPT)

S2 Table Number of copies of HEV RNA detected in cell lysate and supernatant of HEV transfected ΔBISPR Huh7 and Huh7 cells 24 and 72hrs post HEV replicon transfection. (PPT)
