# Supplementary figures and images for: Hepatitis E Virus (HEV) egress: Role of BST2 (Tetherin) and interferon induced long non- coding RNA (lncRNA) BISPR
Source: PLoS One. 2017 Nov 1;12(11):e0187334. doi: 10.1371/journal.pone.0187334 (PMC5665557; doi:10.1371/journal.pone.0187334)

## Slide 1
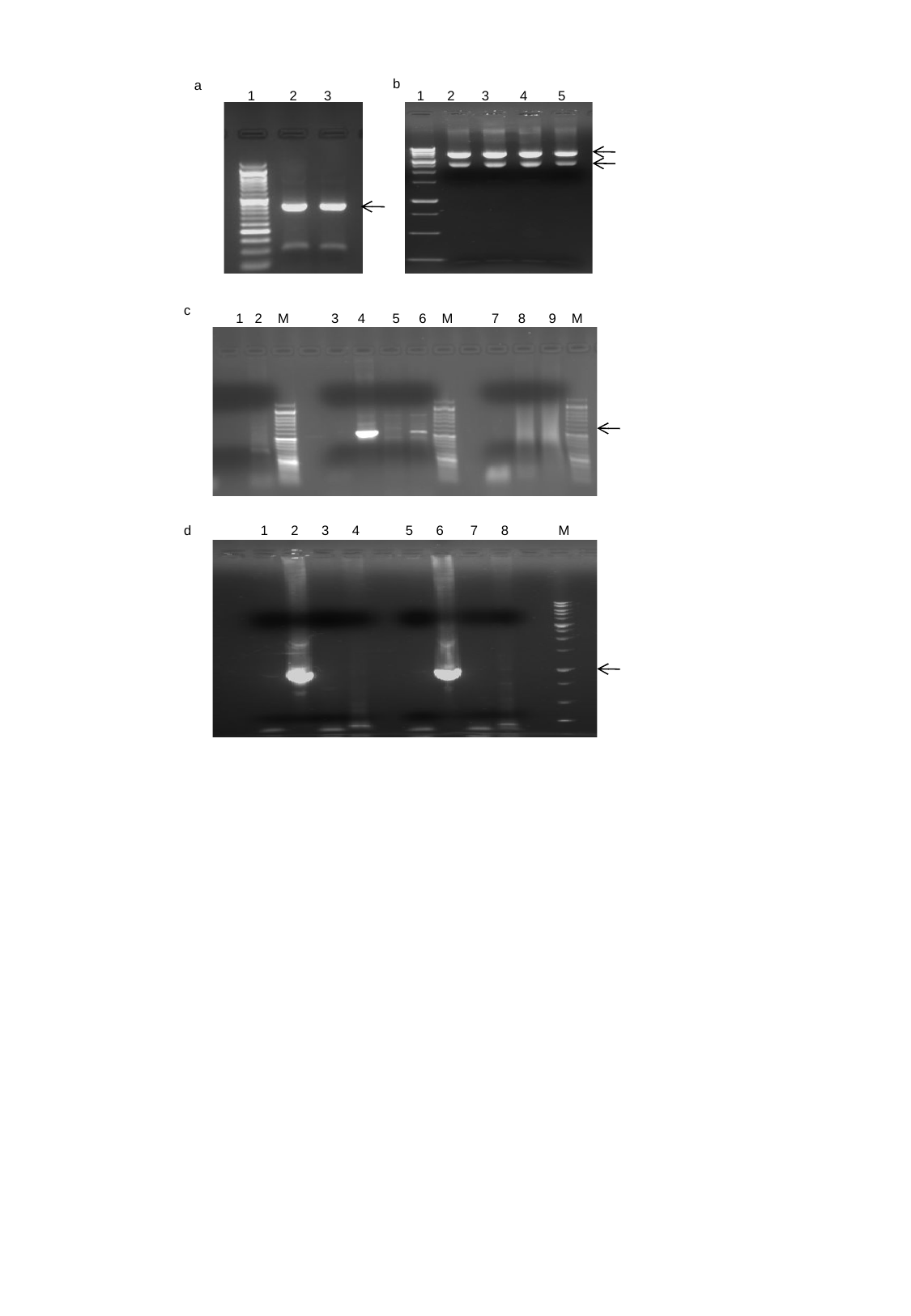

b
a
 1 2 3
1 2 3 4 5
c
 1 2 M 3 4 5 6 M 7 8 9 M
d
 1 2 3 4 5 6 7 8 M

Supplement: S1 Fig — a. 2% agarose gel image representing ~450 bp H1-gRNA1-U6-gRNA2 amplicon generated by fusion PCR (lanes 2 and 3). Lane 1 represents 50bp DNA ladder (BR Biochem, New-Delhi, India).b. 1.2% agarose gel image showing analysis of DNA fragments after EcoRI and NdeI restriction digestion of HR donor vector (with both left and right homology arms ligated in correct orientation). DNA fall-out of ~2.6kb indicates positive constructs (lanes 2–5). Lane 1 shows 1kb DNA ladder (BR Biochem, New-Delhi, India).c. 2% agarose gel image representing PCR based screening of genomic DNA isolated from eight single cell colonies (Huh7) with primers P1and P3 to identify BISPR deletion. Lane 1 represents no template control. Lane 4 and 6 represent colonies positive for BISPR deletion showing ~540 bp amplicon. Lanes marked M represent 50bp DNA ladder (BR Biochem, New-Delhi, India).d. 1.5% agarose gel representing PCR amplification of genomic DNA isolated from eight single cell colonies (Huh7) with HR vector specific primers (BamH1 HR forward and P6 reverse). Lanes 1 and 5 represent no template controls. Lanes 2 and 6 show ~1000bp amplicon from HR donor vector (positive control). No such amplification could be detected in genomic DNA isolated from ΔBISPR Huh7 cells (lanes 3 and 7) and Huh7 cells (lanes 4 and 8). Lane M shows 1kb DNA ladder (BR Biochem, New-Delhi, India). (PPT) [file pone.0187334.s001.ppt]
