# Supplementary material for: Hepatitis E Virus (HEV) egress: Role of BST2 (Tetherin) and interferon induced long non- coding RNA (lncRNA) BISPR
Source: PLoS One. 2017 Nov 1;12(11):e0187334. doi: 10.1371/journal.pone.0187334 (PMC5665557; doi:10.1371/journal.pone.0187334)
Supplement: S2 Fig — a. Profile of control Huh7 cells using blue laser in GFP channel.b. Profile of puromycin resistant Huh7 cells, sorted 14 days post Cas9- gRNA and HR donor vector transfection for high GFP expressing cells.c. Profile of second sort of puromycin resistant Huh7 cells, performed 21days post transfection (7 days after first sort).d. Post sort profile of cells after second sorting (21days post transfection). (PPT) [file pone.0187334.s002.ppt]

## Slide 1
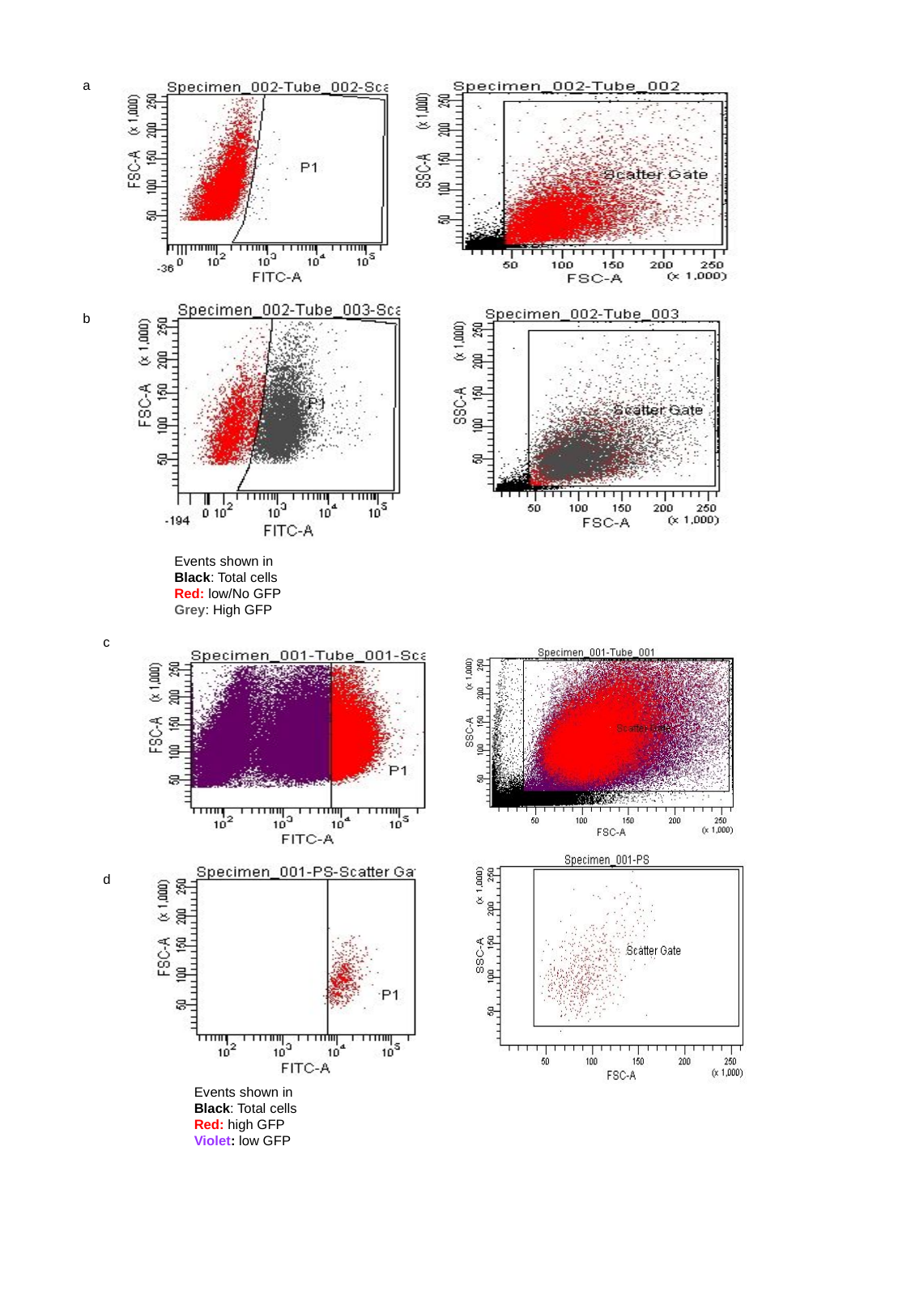

a
b
Events shown in
Black: Total cells
Red: low/No GFP
Grey: High GFP
c
d
Events shown in
Black: Total cells
Red: high GFP
Violet: low GFP
