# Supplementary material for: Hepatitis E Virus (HEV) egress: Role of BST2 (Tetherin) and interferon induced long non- coding RNA (lncRNA) BISPR
Source: PLoS One. 2017 Nov 1;12(11):e0187334. doi: 10.1371/journal.pone.0187334 (PMC5665557; doi:10.1371/journal.pone.0187334)
Supplement: S1 Table — (PPT) [file pone.0187334.s004.ppt]

## Slide 1
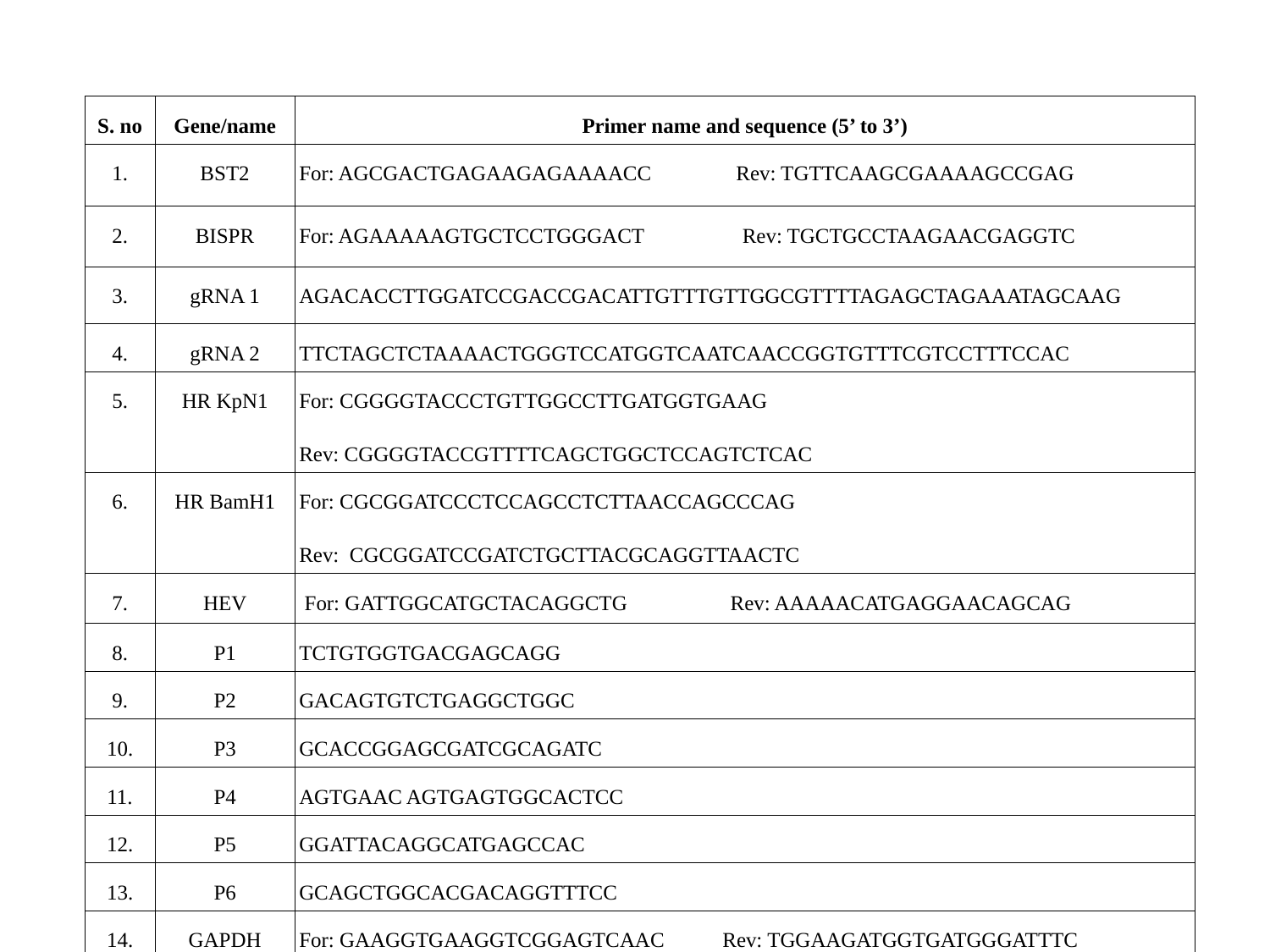

| S. no | Gene/name | Primer name and sequence (5’ to 3’) |
| --- | --- | --- |
| 1. | BST2 | For: AGCGACTGAGAAGAGAAAACC Rev: TGTTCAAGCGAAAAGCCGAG |
| 2. | BISPR | For: AGAAAAAGTGCTCCTGGGACT Rev: TGCTGCCTAAGAACGAGGTC |
| 3. | gRNA 1 | AGACACCTTGGATCCGACCGACATTGTTTGTTGGCGTTTTAGAGCTAGAAATAGCAAG |
| 4. | gRNA 2 | TTCTAGCTCTAAAACTGGGTCCATGGTCAATCAACCGGTGTTTCGTCCTTTCCAC |
| 5. | HR KpN1 | For: CGGGGTACCCTGTTGGCCTTGATGGTGAAG Rev: CGGGGTACCGTTTTCAGCTGGCTCCAGTCTCAC |
| 6. | HR BamH1 | For: CGCGGATCCCTCCAGCCTCTTAACCAGCCCAG Rev: CGCGGATCCGATCTGCTTACGCAGGTTAACTC |
| 7. | HEV | For: GATTGGCATGCTACAGGCTG Rev: AAAAACATGAGGAACAGCAG |
| 8. | P1 | TCTGTGGTGACGAGCAGG |
| 9. | P2 | GACAGTGTCTGAGGCTGGC |
| 10. | P3 | GCACCGGAGCGATCGCAGATC |
| 11. | P4 | AGTGAAC AGTGAGTGGCACTCC |
| 12. | P5 | GGATTACAGGCATGAGCCAC |
| 13. | P6 | GCAGCTGGCACGACAGGTTTCC |
| 14. | GAPDH | For: GAAGGTGAAGGTCGGAGTCAAC Rev: TGGAAGATGGTGATGGGATTTC |
