# Supplementary material for: Hepatitis E Virus (HEV) egress: Role of BST2 (Tetherin) and interferon induced long non- coding RNA (lncRNA) BISPR
Source: PLoS One. 2017 Nov 1;12(11):e0187334. doi: 10.1371/journal.pone.0187334 (PMC5665557; doi:10.1371/journal.pone.0187334)
Supplement: S2 Table — (PPT) [file pone.0187334.s005.ppt]

## Slide 1
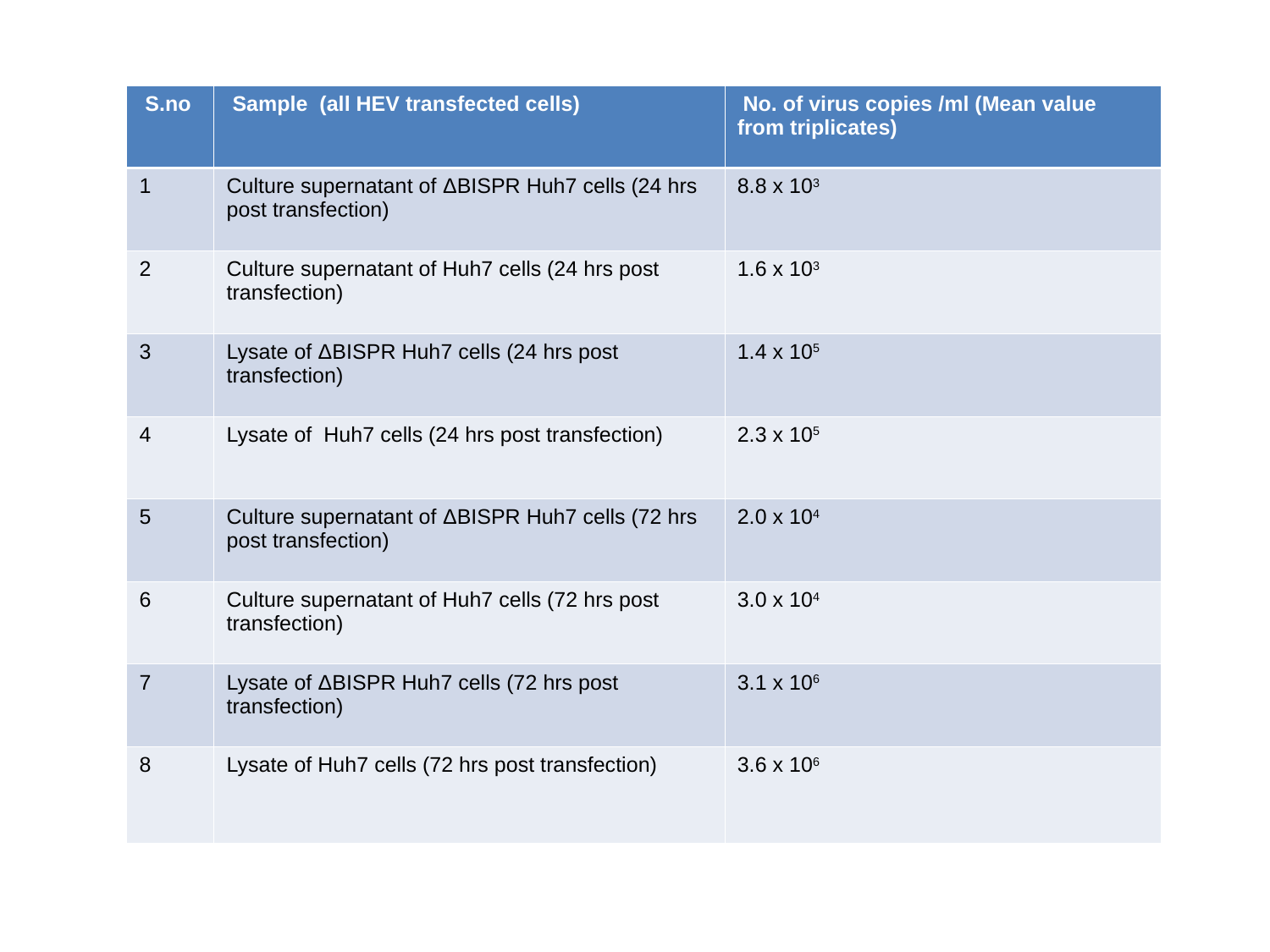

| S.no | Sample (all HEV transfected cells) | No. of virus copies /ml (Mean value from triplicates) |
| --- | --- | --- |
| 1 | Culture supernatant of ΔBISPR Huh7 cells (24 hrs post transfection) | 8.8 x 10^3^ |
| 2 | Culture supernatant of Huh7 cells (24 hrs post transfection) | 1.6 x 10^3^ |
| 3 | Lysate of ΔBISPR Huh7 cells (24 hrs post transfection) | 1.4 x 10^5^ |
| 4 | Lysate of Huh7 cells (24 hrs post transfection) | 2.3 x 10^5^ |
| 5 | Culture supernatant of ΔBISPR Huh7 cells (72 hrs post transfection) | 2.0 x 10^4^ |
| 6 | Culture supernatant of Huh7 cells (72 hrs post transfection) | 3.0 x 10^4^ |
| 7 | Lysate of ΔBISPR Huh7 cells (72 hrs post transfection) | 3.1 x 10^6^ |
| 8 | Lysate of Huh7 cells (72 hrs post transfection) | 3.6 x 10^6^ |
